# MicroRNA-18a promotes proliferation and metastasis in hepatocellular carcinoma via targeting KLF4

**DOI:** 10.18632/oncotarget.19293

**Published:** 2017-07-17

**Authors:** Li Liu, Xun Cai, Enqiang Liu, Xia Tian, Chuan Tian

**Affiliations:** ^1^ Department of Medicine & Appliance, Yunyan District Market Supervision and Administration Bureau, Guizhou 550001, China; ^2^ Department of Oncology, Shanghai General Hospital, Shanghai Jiaotong University School of Medicine, Shanghai 200080, China; ^3^ Department of Oncology, Qianjiang Central Hospital of Chongqing Municipality, Chongqing 409000, China; ^4^ Department of Nuclear Medicine, Guizhou Provincial People’s Hospital, Guizhou 550000, China

**Keywords:** miR-18a, KLF4, p21, hepatocellular carcinoma

## Abstract

MicroRNAs (miRNAs) are short, non-coding and endogenous RNAs that played as important roles in the proliferation and metastasis of tumors. In this study, we determined the role of miR-18a in the regulation of HCC cell motility. We showed that miR-18a expression was upregulated in human HCC tissues and cell lines. Moreover, Elevated expression of miR-18a promoted the HCC cell proliferation and migration. KLF4 was identified as a direct target of miR-18a in HCC cells. Furthermore, overexpression of KLF4 attenuated the effects of miR-18a on the regulation of HCC cell motility. The expression of KLF4 was negatively associated with the expression of miR-18a expression in HCC tissues. We also showed that the cell cycle inhibitor p21 was aberrantly downregulated in HCC cells, whereas this inhibition was reversed by miR-18a inhibitor. These data indicated that miR-18a may play a positive role in hepatocellular carcinoma by promoting the proliferation and migration of HCC cells through targeting KLF4 as well as downstream p21.

## INTRODUCTION

Hepatocellular carcinoma (HCC) is one of the most frequent and most lethal types of cancer worldwide with hardly any effective treatment available thus far [1, 2]. So far, early detection, surgical resection and gene therapy are the major treatment approaches for HCC [3]. Despite significant improvements in diagnostic method and surgical therapy, the cure rate of HCC is very low. Hence, knowledge about the molecular mechanisms underlying HCC progression is an urgent need to improve the understanding of, and therapeutic strategies for, human HCC.

MicroRNAs (miRNAs) are a type of short, non-coding and endogenous RNAs that inhibit the protein coding genes expression through partial complementary binding to the 3’-UTR of mRNAs [4]. Accumulating evidence suggests that the dysregulation of miRNAs is involved in the pathogenesis of multiple human diseases [5–7]. In cancer, Ectopic regulation of miRNA expression has been reported widely and proven to be associated with cancer progression in glioma, metastatic prostate cancer, hepatocellular carcinoma, and others [8, 9]. Various studies also reported the differential expression of miRNAs in patients with hepatocellular carcinoma, including miR-135a, miR-33a, miR-320a, miR-122, and miR-31 [10–14].

Dysregulation of the miR-18a family expression has been detected in various cancers and was proven to be correlated with the biological mechanism of tumor development [15]. miR-18a was found to target the ESR1 gene, which encodes for the estrogen receptor α (ERα) protein. The functional study of the effect of increased levels of miR-18a on both ligand-stimulated transcriptional activation and cell proliferation activity of ERα further supported its involvement in regulating ERα’s functions [16]. In HCC, miRNA-18a was found to be significantly elevated and might be a potential screening biomarker for hepatocellular carcinoma [17]. However, the regulatory mechanism of miR-18a in HCC progression is still unknown.

Krüppel-like factor 4 (KLF4), a zinc-finger trans-cription factor, functions as a tumor suppressor or an oncogene. It plays an important role in cell proliferation and metastasis by regulating the expression of a number of downstream target genes [18]. Accumulating clinical, experimental and mechanistic evidence suggests that KLF4 functions as a tumor suppressor in various types of cancer, including HCC [14]. However, how KLF4 expression is decreased in the progression of HCC is still not well known.

In this study, we found that KLF4 is a potential direct target of miR-18a with a binding site in the 3’-UTR and investigated the role of miR-18a in regulating hepatocellular carcinoma cell proliferation and migration. miR-18a was found to be significantly upregulated in HCC and promotes hepatocellular carcinoma cell motility by inhibiting KLF4. Identification of this mechanism might provide to be a novel understanding and therapeutic approach for HCC.

## RESULTS

### miR-18a expression was upregulated in human HCC tissues and cell lines

**Figure 1:**
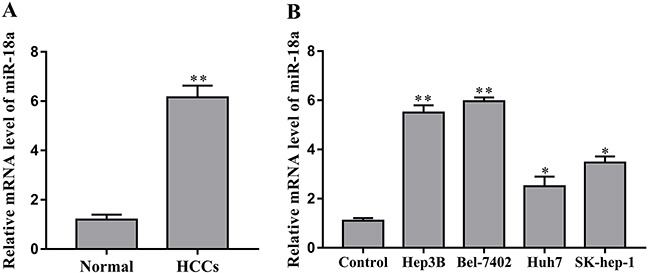
miR-18a is upregulated in human HCC tissues and cells **(A)** The expression of miR-18a was measured in human HCC tissues and adjacent normal liver tissues (normal) by RT-PCR assay (*p < 0.05 vs. normal). **(B)** The levels of miR-18a were examined in human HCC cell lines Hep3B, Bel-7402, Huh7, SK-hep-1 and the normal liver cell LO2 (control) (**p < 0.01,*p < 0.05 vs. control).

We first investigated the expression levels of miR-18a in sample tissues of patients with hepatocellular carcinoma to determine the role of miR-18a in HCC. As shown in Figure 1A, miR-18a was expressed at low levels in the adjacent normal liver tissues, while it was significantly upregulated in the liver cancer tissues.

Furthermore, we investigated the expression of miR-18a in human HCC cell lines Hep3B, Bel-7402, Huh7 and SK-hep-1. Compared to the normal liver cell LO2, miR-18a expression was upregulated in the Hep3B, Bel-7402, Huh7 and SK-hep-1 cells to different extents (Figure 1B). Taken together, these data suggest that miR-18a may play a positive role in the regulation of human HCC progression.

### miR-18a promotes the proliferation and migration of HCC cell lines

**Figure 2:**
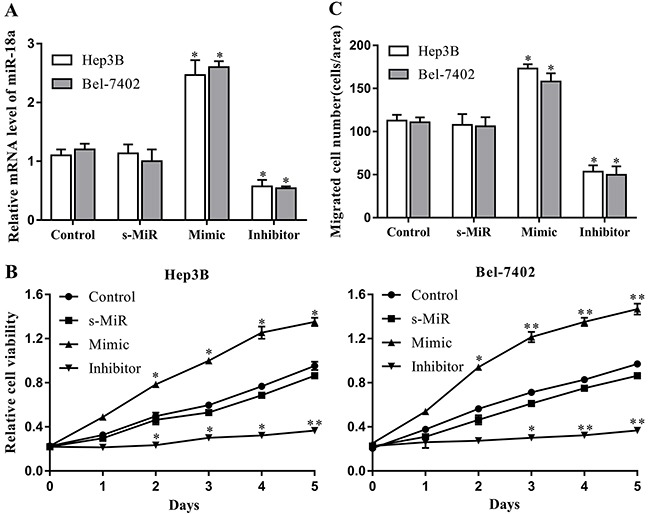
miR-18a promotes the proliferation and migration of human HCC cells Cultured Hep3B and Bel-7402 cells were transfected with miR-18a mimic, inhibitor, or negative control miRNA (s-MiR). **(A)** The levels of miR-18a in cells were evaluated after 48 h of transfection using RT-PCR. **(B)** Cell proliferation was detected at 0-5 days via CCK-8 assays. **(C)** Cell migration was measured after 48 h of transfection by Transwell assay. Nontransfected cells were used as the control (**p < 0.01,*p < 0.05 vs. control).

We then introduced miR-18a mimic and inhibitor to determine the possible effects of miR-18a on the proliferation and migration of human hepatocellular carcinoma cells *in vitro*. Herein, the Hep3B and Bel-7402 cell lines were chosen as the cell models according to the difference of miR-18a expression. The efficiency of miR-18a mimic and inhibitor was confirmed by RT-PCR. Compared with the control miRNA, the miR-18a expression was significantly enhanced by the mimic but reduced by the inhibitor in both Hep3B and Bel-7402 cell lines (Figure 2A).

A CCK-8 assay was performed to examine the effect of miR-18a on the proliferation of Hep3B and Bel-7402 cells. As seen in Figure 2B, miR-18a mimic transfection remarkably increased the proliferation of both Hep3B and Bel-7402 cells compared with the control group. On the contrary, the proliferation capacity of these two cell lines was significantly restrained by miR-18a repression induced by inhibitor. We also confirmed the alteration of Hep3B and Bel-7402 cells migration using Transwell assay. Results showed that the migration of Hep3B and Bel-7402 cell lines was significantly increased by miR-18a overexpression, but repressed by miR-18a silencing (Figure 2C). These results suggest that high levels of endogenous miR-18a may play a regulatory role in the development of HCC by promoting HCC cell proliferation and migration.

### KLF4 is the direct target of miR-18a in hepatocellular carcinoma cells

**Figure 3:**
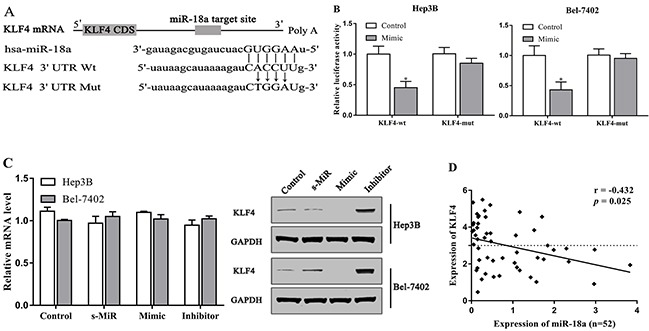
KLF4 is the target of miR-18a in HCC cells **(A)** The predicted binding site of miR-18a in the 3’-UTR of KLF4. **(B)** Luciferase activities were analyzed in the cellstransfected with KLF4-3’-UTR-wt (KLF4-wt) or KLF4-3’-UTR-mut (KLF4-mut). **(C)** The expression of KLF4 in cells was examined via qRT-PCR and Western blot assay. **(D)** The expression of KLF4 was negatively associated with the expression of miR-18a expression in HCC tissues. (*p < 0.05 vs. control).

KLF4 has been considered to be a tumor suppressor in HCC by previous studies [19]. To further elucidate the underlying mechanism of miR-18a regulating hepatocellular carcinoma cells, we predicted the possible targets of miR-18a using TargetScan 6.2 and miRDB databases. To further confirm whether or not this prediction is right, we performed the luciferase reporter assay in Hep3B and Bel-7402 cells. The 3’-UTR regions of KLF4 containing the predicted binding site of miR-18a or the mutant site were cloned into a luciferase vector (Figure 3A). It was found that miR-18a overexpression significantly inhibited the luciferase activities of KLF4-3’-UTR-wt reporter in two cell lines, whereas miR-18a mimic transfection exhibited no inhibitory effects on the luciferase activities of KLF4-3’-UTR-mut reporter in cells (Figure 3B).

In addition, we confirmed that the protein level of KLF4 was strongly downregulated by miR-18a mimic, but increased by miR**-**18a inhibitor in Hep3B and Bel-7402 cells (Figure 3C). However, there was no effect of miR-18a mimic and inhibitor on the mRNA expression of KLF4. The expression of KLF4 was negatively associated with the expression of miR-18a expression in HCC tissues (Figure 3D).

### miR-18a serves as a positive regulator of HCC cell motility by targeting KLF4

**Figure 4:**
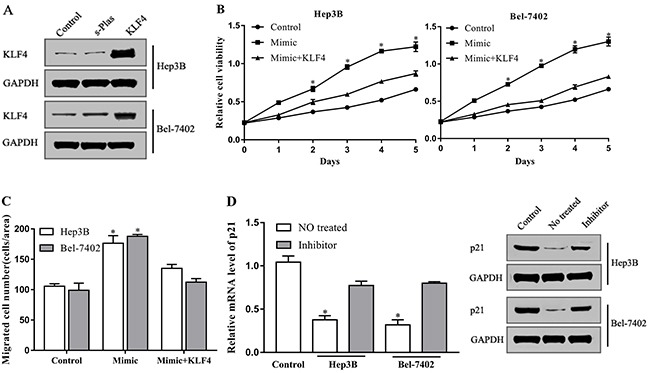
miR-18a increases HCC cells motility by targeting KLF4 **(A)** The protein levels of KLF4 were detected via Western blot in Hep3B and Bel-7402 cells transfected with KLF4 plasmid or negative control plasmid (s-Plas). After cotransfection with miR-18a mimic and KLF4 plasmid, **(B)** cell proliferation and **(C)** migration were then tested. **(D)** The level of p21 in Hep3B, Bel-7402, and LO2 cells (control) was measured by RT-PCR and Western blot assay. (*p < 0.05 vs. control).

miRNAs play a role in the regulation of cellular function by inhibiting target genes. To explore that KLF4 is indeed the downstream mediator of miR-18a in promoting hepatocellular carcinomacell motility *in vitro*, we employed a KLF4 plasmid to specifically induce the expression of KLF4 in cells (Figure 4A). The upregulation of KLF4 expression reversed the promoting effect of miR-18a on the proliferation capacity of Hep3B and Bel-7402 cells (Figure 4B). The migration of cells induced by miR-18a mimic was also inhibited by KLF4 overexpression as shown in Figure 4C, indicating that miR-18a promotes the proliferation and migration of hepatocellular carcinoma cells by targeting KLF4.

Cell cycle inhibitor p21 is a downstream signal molecule of KLF4. Previous studies indicate that KLF4 inhibits cellular proliferation and migration by directly targeting p21 [20]. To further confirm the involvement of KLF4 in miR-18a-mediated effects, we investigated the expression of p21. Compared with the normal liver cell LO2, p21 expression was downregulated in Hep3B and Bel-7402 cells, whereas this inhibition was reversed by miR-18a inhibitor (Figure 4D). These findings imply that miR-18a plays a positive role in the regulation of hepatocellular carcinoma cell motility by repressing the inhibitory effect of KLF4 on cell cycle progression.

## DISCUSSION

Differential expression of microRNAs in patients with hepatocellular carcinoma was reported in numerous studies, such as miR-135a, miR-33a, miR-320a, miR-122, and miR-31 [10–14]. Although over or underexpression of microRNAs occurring in HCC has been considered to contribute to hepatocellular carcinoma, the potential mechanism by which miRNAs regulate hepatocellular carcinoma progression needs to be further explored. Previous reports demonstrate that miRNA-18a expression is significantly upregulated in HCC and may present a novel screening biomarker for early diagnosis of HCC [17]. However, the precise role of miR-18a in the pathogenesis of HCC remains unknown. In the present study, we determined that miR-18a is aberrantly upregulated in HCC and promotes hepatocellular carcinoma cell motility by inhibiting KLF4, implying that miR-18a may play a positive role in the regulation of human HCC progression.

Emerging evidence indicates that cell malignant proliferation and invasion are the main causes that contribute to tumorigenesis and carcinogenesis in humans [21]. In HCC, it is reported that dysregulation of miRNAs has diverse effects on hepatocellular carcinoma cell motility. miR-765 promotes cell proliferation by downregulating INPP4B expression in human hepatocellular carcinoma [22]. Yuan et al. also reported that MicroRNA-340 is significantly downregulated in HCC tissues and can suppress the proliferation of HCC cells by directly targeting JAK1 [23]. In addition, our results revealed the positive role of miR-18a in HCC tumorigenesis. Increased expression of miR-18a increased the proliferation and migration capacity of cultured HCC cells, whereas miR-18a repression exhibited an inhibitory effect on HCC cell motility *in vitro*.

KLF4 is a member of KLF zinc finger transcription factor family that is enriched in a variety of tissues with a critical role in the regulation of cell differentiation and development. Accumulating evidence determined that KLF4 is expressed at a low level and exerts a tumorsuppressive effect in many types of cancer [10, 14, 18, 19]. In HCC, KLF4 expression is also remarkably downregulated in HCC tissues compared with matched normal tissues [19]. Yao et al. reported that KLF4 has an effect as a target of miR-135a-5p on the modulation of TGF-β1 to hepatocellular carcinoma metastasis by regulating the expression of KLF4 [10]. Here, we demonstrated that KLF4 is a direct target of miR-18a and can be inhibited by miR-18a at the posttranscriptional manner in HCC cells. Notably, we found that the overexpression of KLF4 reverses the effect of miR-18a on the proliferation and migration of HCC cells, indicating that miR-18a may increase the motility of HCC cells by directly targeting KLF4.

As the downstream target of KLF4, p21 has been identified as a negative regulator in cell cycle progression. It is reported that p21 transactivation is induced by the binding of KLF4 to its promoter, contributing to the inhibitory role of p21 in cell cycle [24]. We investigated the effects of miR-18a-KLF4 on downstream p21 in HCC cells. Compared with normal liver cell, p21 expression is significantly downregulated in cultured HCC cells, which is consistent with the findings in previous studies [25]. In contrast, miR-18a inhibitor significantly increased the expression of p21 in parallel with an enhanced KLF4 level, indicating that miR-18a may promote hepatocellular carcinoma cell growth and invasion by regulating the activity of KLF4 as well as downstream p21.

In summary, we provide a novel understanding about the pathogenesis of human HCC. Increased miR-18a level plays a positive role in hepatocellular carcinoma by promoting the proliferation and migration of HCC cells through targeting downstream KLF4 and p21. This may offer an alternative therapeutic target for HCC.

## MATERIALS AND METHODS

### Samples and cells

Fifty-two HCC tissue specimens were collected from patients who had undergone complete surgical resection at the Department of Hepatic Surgery, the Eastern Hepatobiliary Surgery Hospital of Shanghai Second Military Medical University (Shanghai, China) between June 2015 and August 2016, immediately snap-frozen in liquid nitrogen and stored at **-**80°C. All tissue specimens (both HCC tissues and adjacent non-cancerous tissues) were constructed for experiments at the same time. Moreover, all patients provided their written informed consent and the hospital ethical committee approved the experiments.

Human HCC cell lines Hep3B, Bel-7402, Huh7 and SK-hep-1 were obtained from the Shanghai Cell Bank of Chinese Academy of Sciences (Shanghai, China). The liver cell line, LO2, was purchased from Li Yandong Research Group Shanghai East Hospital affiliated to Tongji University School of Medicine. Each cell line was cultured in Dulbecco’s modified Eagle medium (DMEM, Invitrogen, USA) supplemented with 10% fetal bovine serum (FBS, Hyclone) as well as 100 u/ml penicillin and 100 ug/ml streptomycin. All of the cells were maintained in a humidified incubator at 37°C with 5% CO_2_.

### Quantitative RT-PCR

Trizol reagent (Invitrogen, Carlsbad, CA, USA) was used to isolate the total RNA from tissues and cells. Expression of miR-18a was measured using MicroRNA First-Strand Synthesis and miRNA Quantitation kits (Takara, Dalian, China) according to the manufacturer’s instructions. CellAmp Direct RNA Prep kit for qPCR and a Protein Analysis kit (Takara) were used to detect the KLF4 and p21/CIP1 expression. The CT values of U6 and GAPDH were used as the internal control to normalize the relative expression of miR-18a and KLF4 respectively. The reaction was performed as follows: 10 min at 95°C; 40 cycles of 1 min at 95°C, 2 min at 63°C, 1 min at 72°C; final annealing at 72°C for 10 min. The differential expression level was calculated using the 2^−ΔΔCT^ formula. All PCRs were performed in triplicate.

### Western blotting

The HCC cells were lysed and extracted into protein with 1× SDS–PAGE loading buffer. Protein concentrations were determined using the BCA protein assay kit (Beyotime Institute of Biotechnology, China). Equal amounts of protein were separated on 10% SDS-PAGE gel and then transferred to the PVDF membranes. After blocking with 5% skim milk for 2 hours, the membranes incubated with primary antibodies as follows: KLF4 (1:500, Santa Cruz Biotechnology) and GAPDH (1:3000, Santa Cruz Biotechnology).

### Cell transfection

The miR-18a mimic, inhibitor, and negative control miRNA were purchased from RiboBio (Guangzhou China) and transfected into cells at 100 nM concentrations via Lipofectamine 2000 (Invitrogen) according to the manufacturer’s instructions. KLF4 plasmid (1ug; Origene, Rockville, MD, USA) was also transfected into cells in the presence or absence of the miR-18a mimic by Lipofectamine 2000. After transfection twice in 48 h, cells were used in the subsequent experiments. RT-PCR and Western blotting were used to evaluate the transfection efficacy.

### Cell proliferation assay

Cell proliferation was determined by Cell Counting Kit (CCK-8/WST-8) (Bioroot, Shanghai, China) according to the kit’s instructions. Briefly, 10 ul of CCK-8 solution was added to the transfected cells that were planted in the 96-well plates (5×10^3^ cells/ml) at days 0-5 and incubated for 4 h at 37°C. The absorbance of cells was detected at 450 nm using ELISA plate reader (Bio-tek, Winooski, VT, USA).

### Cell migration assay

Cell migration was measured in the Transwell Boyden chamber. Briefly, after 2 days of transfection, cells were incubated in the growth factor-free DMEM and then moved into the upper chamber of Boyden chambers coated with gelatin. The lower compartment of the chamber was filled with 600 ul of DMEM medium supplemented with 10% FBS. Cells were incubated at 37°C for 4 h, followed by fixation with 90% ethanol and staining with 0.05% crystal violet for 15 min. Nontransmigrated cells were gently scraped off using cotton swab. Migrated cells were determined by counting the cells on the lower membrane surface within five fields per chamber under a microscope.

### Luciferase assay

Site-directed mutagenesis was introduced into the miR-18a binding site of KLF4 mRNA using QuikChange Lightning Site-Directed Mutagenesis Kit (Stratagene). The 3’-UTR fragment of KLF4 mRNA was then subcloned into the pGL3 luciferase vector (Promega, Madison, WI, USA) by PCR method and cotransfected with miR-18a mimic into HCC cells for 36 h in 96-well plates using Lipofectamine 2000. Dual Luciferase Assay (Promega) was then performed to analyze the luciferase assays. Renilla (Promega) activity was used as the internal control.

### Statistical Analysis

All data are expressed as mean ± SEM. The differences between groups were analyzed using SPSS 19.0 by one-way ANOVA. A value of *p* < 0.05 was considered statistically significant. All experiments were performed in triplicate.
